# Acknowledgement to Reviewers of *Pharmacy* in 2019

**DOI:** 10.3390/pharmacy8010012

**Published:** 2020-01-20

**Authors:** Pharmacy Editorial Office

**Affiliations:** MDPI, St. Alban-Anlage 66, 4052 Basel, Switzerland

The editorial team greatly appreciates the reviewers who have dedicated their considerable time and expertise to the journal’s rigorous editorial process over the past 12 months, regardless of whether the papers are finally published or not. In 2019, a total of 162 papers were published in the journal, with a median time to first decision of 16 days and a median time from submission to publication of 43.5 days. The editors would like to express their sincere gratitude to the following reviewers for their generous contribution in 2019:
Abou-Ali, AdelKovvasu, Surya PrakasaraoAhmed, ZehraKrause, Jane E. Alderman, ChrisLavergne, Sidonie N.Alegro, JasonLee, Chooi YengAlessandri, ClaudiaLeng, WichitahAlipour, AzitaLiu, YifeiAllegaert, KarelLongyhore, Daniel S.Anderson-Haag, TracyLonie, John M.Andreski, MichaelLópez-López, DanielArnett, RichardLucas, MartinAshiru-Oredope, DianeMachado, Humberto S.Athinarayanan, ShaminieMacLure, KatieAustin, ZubinMadzhidova, ShirinAyers, PhilMaggo, SimranBagiu, Iulia-CristinaManchia, MirkoBaker, DanialManning, Dana H.Baqir, WasimMantzourani, EfiBarnard, MarieMarston, LouiseBarnett, NinaMarvanova, MarketaBarton, GarryMathieson, StephanieBaumgarden, JosephMayberry, KatelynnBauters, TieneMaynard, GreggoryBell, HeatherMcAuley, JamesBelousova, VictoriaMcDonough, RandyBennett, Jill HinesMcGee, Edo-Abasi U.Berger, JérômeMestres, ConxitaBerry, TriciaMestrovic, ArijanaBhattacharjee, SonaliMihalopoulos, CleopatraBingham, JennyMira, José JoaquínBosnic-Anticevich, SinthiaMixon, AmandaBožič, BorutMolina-Mula, JesúsBragazzi, Nicola LuigiMorales, Ana IsabelBrown, Lawrence M.Morgan, JillBrunetti, LuigiMorrissey, HanaBryniarski, KrzysztofMuceniece, RutaBuda, Valentina OanaMurzello, AndreaBuerki, RobertNajib, Jadwiga S.Bungau, Simona GabrielaNarayan, SujitaCaballero, PabloNash, CarolCaduff-Janosa, PiaNemecek, BrandenCarboni, LuciaNielsen, NicoleCavaco, Afonso M.Norman, Elizabeth J.Cavé, ChristianNørskov-Lauritsen, NielsCavicchi, CaterinaNounou, Mohamed IsmailChang, JongwhaObreza, AlešChastain, DanielOhji, GohChatterjee, SatabdiOligbu, GodwinChaudhri, VirendraOrtega-Ávila, Ana BelénChen, Chieh-FuOsowiecka, KarolinaChilds-Kean, LindseyOttis, EricaChng, Hui TingOwens, ChristopherColey, Kim C.Pai, AmyCostantino, ClaudioPalombi, LauraCovington, Elizabeth W.Park, KatharineCrawford, Stephanie Y.Parlesak, AlexandrCropp, CherylParrish II, Richard H.Culhane, JamesPatel, NileshCurran, GeoffreyPathak, AshishDang, RichardPatman, ShaneDaverey, AmitaPatyra, EwelinaDawoud, DaliaPaudyal, VibhuDe Loof, HansPeletidi, AlikiDe Rosis, SabinaPerez-Inestrosa, EzequielDemirkol, ApoPerpelkin, JasonDering-Anderson, AllyPeterson, GregoryDeshpande, MaithiliPettit, NatashaDestache, Christopher J.Porebski, GrzegorzDhital, RanjitaPotter, KathleenDiack, LesleyPowers, James S.Dibaba, DanielPrzybyłek, MaciejDietrich, EricPuglisi-Weening, MelanyDonald, Bryan J.Rahman, AteequrDonyai, ParastouRahmanpour, RahmanDovinova, ImaRamachandran, SujithDrovandi, AaronRamos, VictoriaDugan, B. DeeAnnRascher, WolfgangDuong, PhuRather, Irfan AhmadDurham, SpencerRavula, AbhigyanDutour, OlivierRefai, DeemaEhret, Megan J.Regiec, AndrzejEid, DeebRichardson, Carolyn M.Eppard, ElisabethRoche, CicelyFelgueiras, HelenaRosenthal, MeagenFerreri, StefanieRutter, PaulFinley, BrentRyder, SheilaFlood, MichelleSadowski, Cheryl A.Ford II, James H.Sakharkar, PrashantFouladkhah, AliyarSánchez-Pozo, AntonioFuji, KevinSarpong, Eric M.Furneri, Pio MariaSaura, José RamónGadbois, Natalie R.Schein, RebeccaGala, Rikhav P.Schmidt, Jonas DamgårdGallagher, PaulSchneider, JenniferGallego, GisselleSchofield, PatriciaGernant, Stephanie A.Schommer, JonGifford, AlisonScott, Mollie AsheGiles, AmberSeed, Sheila MGionfriddo, Michael R.Shaeer, KristyGolchin, NegarShakya, AkhileshGonçalves, José António FerrazSharma, ManviGonzalez-Alvarez, IsabelSimpson, MareeGoode, Jean-VenableSjölander, MariaGray, Jeffrey A.Skowron, AgnieszkaGregorio, JoaoSlimano, FlorianGrieve, LorinSmith, MeganGudka, SajniSobocińska, MagdalenaGuirguis, AmiraSpooner, S. AndrewHammerl, Jens AndreSporrong, Sofia KälvemarkHan, JayoungStehlik, PaulinaHan, ZheStranges, Paul M.Hanna, Lezley-AnneSun, WujinHarley, DavidTambe, MitaliHawboldt, JohnTan, IsabellaHersberger, Kurt E.Teschke, RolfHeslop, IanThompson, JessicaHigby, Gregory J.Tonelli, MarkHillman, LisaTook, RoxaneHilton, Thomas F.Torres-Sangiao, EvaHitch, GeetaTrinh, TrangHo CM, RogerUm, IreneHodurski, TraceyUnni, Elizabeth J.Hohmeier, Kenneth C.Urick, BenHollingworth, SamanthaVaismoradi, MojtabaHoule, SherilynVeve, Michael P.Huang, AprilVlahu-Gjorgievska, ElenaHultgren, KyleVliagoftis, HarissiosHurley-Kim, KeriWatkins, KimIorga, MagdalenaWenzler, Eric R.Jacobs, David M.Wertheimer, AlbertJacobsen, RamuneWietholter, Jon P.Jalal, ZahraWilby, KyleJalava, KatriWolever, Ruth Q.Janikowska, GrażynaWright, Daniel F. B.Jayakumar, RebeccaWright, DavidKaae, SusanneWu, WenchenKatajavuori, NinaXu, GuangKedrowicz, AprilYadav, DhananjayKeers, RichardYamamura, ShigeoKelliher, FelicityYancey, AbigailKelly, FionaYang, JiminKennedy, Mary-ClaireYap, Kai ZhenKheir, NadirYeung, EugeneKirk, LorenYin, JunKnapp, Katherine K.Yunusa, IsmaeelKosari, SamZelkó, RománaKoster, AndriesZhu, Jingen

